# Comparative Study of Pd/B_4_C X-ray Multilayer Mirrors Fabricated by Magnetron Sputtering with Kr and Ar Gas

**DOI:** 10.3390/ma13204504

**Published:** 2020-10-11

**Authors:** Hangjian Ni, Qiushi Huang, Genchang Liu, Runze Qi, Zhong Zhang, Xiuhong Li, Zhongliang Li, Jie Wang, Zhanshan Wang

**Affiliations:** 1 Key Laboratory of Advanced Micro-Structured Materials MOE, Institute of Precision Optical Engineering, School of Physics Science and Engineering, Tongji University, Shanghai 200092, China; nhj2365@163.com (H.N.); liugenchang@tongji.edu.cn (G.L.); qrz@tongji.edu.cn (R.Q.); zhangzhongcc@tongji.edu.cn (Z.Z.); wangzs@tongji.edu.cn (Z.W.); 2 Shanghai Advanced Research Institute, Chinese Academy of Sciences, Zhangheng Road 239, Shanghai 201204, China; lixiuhong@zjlab.org.cn (X.L.); lizhongliang@zjlab.org.cn (Z.L.); wangjie@zjlab.org.cn (J.W.)

**Keywords:** Pd/B_4_C, multilayer, interface quality, heavy noble gas, hard X-ray

## Abstract

Ultrathin Pd/B_4_C multilayers are suitable X-ray mirrors working at the photon energy region of 7–20 keV. To further improve the layer structure, Pd/B_4_C multilayers with a d-spacing of 2.5 nm were fabricated by magnetron sputtering using the heavy noble gas Kr and compared with the conventional ones fabricated by Ar. Although the Kr-sputtering process can work at a lower pressure, the interface width—especially the interface roughness—is a little larger than that made by Ar. A stronger polycrystallization and a lower content of sputter gas atoms were found in the Kr-made sample, which can be explained by the joint effect from less recoiled particles and lower sputtering pressure. A good reflectance of 68% of the Kr made multilayer was measured at 10 keV, which is only slightly lower than that of the Ar made sample (71%).

## 1. Introduction

Multilayer interference coating is an important optics in the extreme ultraviolet and X-ray region. It enables the reflection of the short-wavelength light beyond the total external reflection region, and selects a narrow bandwidth from the incoming spectrum. Thus, it is widely used in various imaging, spectroscopy, and monochromator systems for advanced photolithography, in synchrotron radiation facilities, for astronomical observation, and in plasma diagnostics [1]. As the photon energy increases to the hard X-ray region (e.g., required in synchrotron radiation facilities or space telescopes), the short wavelength requires an extremely small d-spacing of less than 3 nm. This makes the X-ray reflectance very sensitive to the interface quality of the multilayer. Magnetron sputtering has been used as a main fabrication technique after decades of development, thanks to the higher energy of the deposited atoms than in evaporation, the good controllability of the process, and the relatively low cost of the instrument [2]. It is also widely used for making other multilayer films [3,4]. To further improve the interface quality, different interface engineering methods based on physical sputtering were developed. These include the diffusion barrier layer [5,6], reactive sputtering with a mixture of gases [7,8], assisted ion polishing [9,10], and so on. Different methods have been used to deal with different interface problems, such as inter-diffusion, chemical reaction, or interfacial roughness, for specific multilayer systems.

Besides the abovementioned methods, the sputtering gas (the species bombarding the target) is another important factor affecting the deposition process. The atomic mass of the sputtering gas can significantly change the sputtering yield and bombarding effect on the substrate. Argon is the most widely used sputtering gas due to its medium mass and low cost. Generally, a heavier noble gas can have larger ionization cross section and work at lower sputtering pressure [11,12]. The low pressure can reduce the collision probability and increases the kinetic energy of atoms arriving on the substrates. The atoms sputtered by heavier ions can also have a higher ejection energy [13]. These enhance the diffusion mobility of the adatoms and help to form a smooth growth of layers, especially at small thicknesses [14]. Maeda et al. reported that the surface roughness of Co/Pt multilayers, fabricated by magnetron sputtering with Xe, was reduced from 0.31 nm to 0.20 nm as the pressure decreased from 0.47 Pa to 0.09 Pa [12]. On the other hand, part of the bombarding effect during deposition is from the sputtering gas atoms recoiled from the targets. The energy of the recoiled neutrals is related to the mass difference between the gas and the target. Carcia et al. simulated the energy distribution of the recoiled Ar, Kr, and Xe atoms from a Pt target, assuming an incident energy of 500 eV. The peak energy of Kr and Xe reflected neutrals was approximately 2–3 times smaller than that of Ar [15]. It has been demonstrated that the lower energy of the recoiled heavy neutrals can mitigate the intermixing that occurs at the interfaces, as observed in Co/Pt and Gd/Fe magnetic multilayers [2]. A similar effect was also indicated in the growth of Mo/Si multilayers and that the sputtering of Xe gas resulted in a smaller interdiffusion at the Mo-on-Si interface than Ar [16]. Nevertheless, the weaker bombarding effect of the recoiled atoms can also lead to a larger layer roughness. Tang et al. found a rougher surface morphology of Co/Pt multilayers deposited by Kr and Xe as compared with Ar [17]. Maeda et al. reported that the surface roughness of Co/Pt multilayers was 0.14 nm, 0.24 nm, and 0.31 nm as fabricated by Ar, Kr, and Xe gas, respectively [12]. The balance between the interdiffusion and roughness makes the effect of sputtering with heavier gases complicated, which needs to be studied specifically for different material systems.

Hard X-ray multilayers are widely used as monochromator optics in synchrotron radiation facilities. Compared to the crystal monochromator, a multilayer monochromator can provide one to two orders magnitude higher integral flux with a moderate energy resolution [18]. Pd/B_4_C is one of the ideal multilayer candidates, with high theoretical reflectance working in the 7–24 keV region, as both materials have no absorption edges in this range [19]. Other multilayers, such as Mo/B_4_C and Ni/B_4_C [20], cannot work over this whole range given that the absorption edges of Mo and Ni are at 20 keV and 8.3 keV respectively. Although the Pd/B_4_C multilayer fabricated by Ar showed relatively small interface widths [18], a significant drop in experimental reflectivity compared to the theoretical value still exists, especially after double reflections as used in the monochromator. The reactive sputtering technique using a mixture of Ar and N_2_ gases was tested on Pd/B_4_C multilayer, resulting in a greater interface roughness [21]. According to our knowledge, the sputtering process using heavier gases than Ar has not been reported on hard X-ray multilayers. Previous research works were mainly focused on the development of magnetic multilayers, and only a few works were made on Mo/Si multilayers for extreme ultraviolet (EUV) lithography [16,22]. The materials combinations for different applications are quite different, and the sputtering effects on the final magnetic or optical performance are also different, even between the EUV and X-ray region. To further improve the interface quality and reflectivity of Pd/B_4_C multilayers and to understand the sputtering effects of heavy noble gas on small d-spacing X-ray multilayers, a comparative study of Pd/B_4_C multilayers fabricated by Ar and Kr gases was performed in this paper. The interface structure, layer morphology, and X-ray reflectance of the multilayers were investigated and discussed systematically.

## 2. Materials and Methods

**Figure 1:**
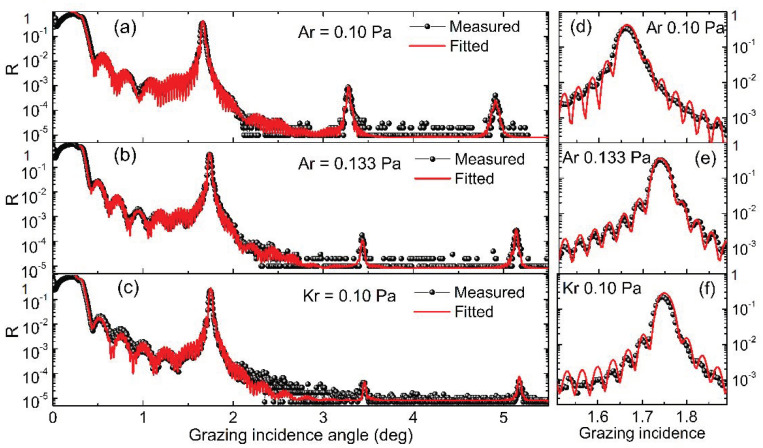
Grazing incidence X-ray reflectometry (GIXR) measurement and fitted results of the Pd/B_4_C multilayers with 50 bilayers fabricated by Ar at 0.10 Pa (**a**,**d**), Ar at 0.133 Pa (**b**,**e**), and Kr at 0.10 Pa (**c**,**f**).

The Pd/B_4_C multilayer was designed to work in the 7–15 keV region with a d-spacing of 2.5 nm. The thickness ratio, γ = d_Pd_/d, was set at 0.5 in order to achieve high harmonics suppression. Both multilayers with a number of bilayers of N = 50 and 150 were fabricated, and N = 150 is the saturated value for maximum reflectance. All multilayers were fabricated by direct current magnetron sputtering using a linear deposition system [23]. The system can deposit a maximum mirror with 1.2 m length and 0.2 m width. During deposition, the substrate was moved across the sputtering area of each target for the coating of one layer. High-purity argon (99.999%) and krypton (99.997%) were used as sputtering gases. The lowest stable working pressures were used for Ar and Kr, 0.133 Pa and 0.10 Pa, respectively. The detailed comparison can be seen in Figure 1. The base pressure before deposition was approximately 8.5 × 10^−5^ Pa. Super-polished Si (100) wafers with a root-mean-square roughness of <0.2 nm were used as substrates, with a size of 20 mm × 40 mm. The material purity of sputtering targets of Pd and B_4_C were 99.99% and 99.5%, respectively.

The fabricated multilayers were characterized by grazing incidence X-ray reflectometry (GIXR) at 8.05 keV using a lab-based X-ray diffractometer (Bede, Durham, UK). The incident X-ray beam experienced double reflections from an Si (220) crystal to provide high monochromaticity, and the angular divergence of the beam was approximately 0.007°. The accurate hard X-ray reflectance of the multilayers with 150 bilayers was measured at 10 keV, at the X-ray test beamline (BL09B) in the Shanghai Synchrotron Radiation Facility (SSRF), China [24]. In addition to the double-crystal monochromator, an extra channel-cut crystal optics was added in front of the multilayer sample during the measurement to reduce the beam divergence down to approximately 0.001°.

The reflectivity curves were fitted by IMD software using a simple two-layer model to analyze the layer structure [25]. The error function was used as the interface profile function to account for the effect of the interface roughness and diffusion on reflectance. A systematic layer thickness drift from the bottom to top of the multilayer was taken into account in the layer model to fit the slightly broadened peaks while the interface widths remained the same for all bilayers. Based on the GIXR fitted models, the depth distribution of scattering length density and the refractive index of the layer structures were calculated using GenX software (GenX-2.4.10-win) [26]. To compare the interface roughness, scattering measurements using rocking curve scan were performed on the 150-bilayer samples made by Ar and Kr. The rocking curves were measured by scanning the sample stage around the first Bragg peak position while the sum of the incident and scattering angles remained unchanged. High-resolution transmission electron microscopy (TEM) measurements were made to compare the microstructure and layer morphology of the two samples with 150 bilayers. The samples were prepared by focused ion beam and observed by FEI Talos F200X (FEI, Hillsboro, OR, USA). The elemental composition of the layers was further measured by electron energy loss spectroscopy (EELS) during the TEM measurements. One-dimensional line scans across several bilayers were performed to measure the composition depth profiles of different elements.

## 3. Results and Discussion

**Figure 2:**
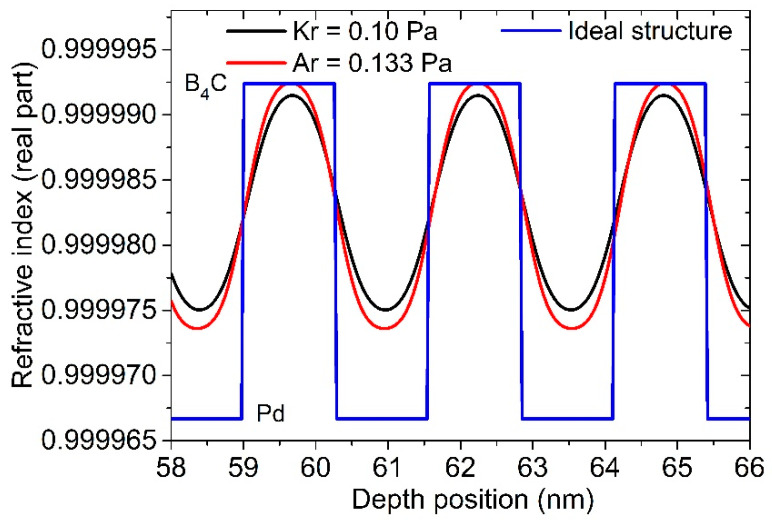
Reconstructed refractive index profiles of the multilayers sputtered by Kr and Ar.

The GIXR results of the 50 bilayer multilayers fabricated by Ar and Kr are shown in Figure 1. The fitted structural parameters are listed in Table 1. The multilayer deposited by Ar at 0.10 Pa showed broadened Bragg peaks (Figure 1a,d) which was caused by an obvious drift of d-spacing over the 50 bilayers. The fitted result indicates a total drift of 60 pm from the bottom to the top of the stack, reaching 2.2% of the d spacing. Considering the thickness drift, the average layer thickness of each material is given in Table 1. The average interface width of the multilayer was 0.27 nm. After increasing the pressure of Ar to 0.133 Pa, the Bragg peaks became narrower and the drift significantly reduced to 30 pm (Figure 1b,e). The average interface width was 0.26 nm, which is similar to the one deposited under lower pressure. It is evident that the Ar sputtering process is not sufficiently stable under the pressure of 0.10 Pa. The multilayer deposited by Kr at 0.10 Pa showed narrow peaks with a total drift of only 25 pm. This proves the lower stable working pressure of the Kr sputtering process compared to that of Ar [1]. However, the fitted interface width was 0.30 nm, which is a little larger than the one produced by Ar. This can be clearly seen by the diminished second- and third-order peaks (Figure 1c). Both samples showed slightly lower density of the Pd layers, especially for the Kr-sputtered one which was lower than the Ar-sputtered sample. The depth distribution of the refractive index (real part) of the two multilayers deposited by Ar (0.133 Pa) and Kr (0.10 Pa) was calculated based on the fitted model, as shown in Figure 2. The index of Pd and B_4_C layers with bulk density (12.02 g/cm^3^ and 2.52 g/cm^3^, respectively) was also added as a comparison. The index profile gradually varied with no plateau inside each layer, indicating that there was no pure Pd or B_4_C layers in the stack. This can be explained by the intermixing of the two materials under the ultrasmall thickness. The gradual variation of the index profile is represented by the interface width and layer density in the two-layer model used above for fitting. The amplitude of the index variation of the Kr-sputtered sample was slightly lower than that of the Ar-sputtered sample, indicating a lower optical contrast of the multilayer, which will reduce the reflectance.

Another noticeable difference was the growth rate of Pd and B_4_C layers sputtered by two gases. The growth rate of Pd on the substrate using Kr was slightly increased by a few percent compared to Ar, while the rate of B_4_C was dramatically reduced by around 40%. This difference could be caused by the change of sputtering yield and deposition probability (the ratio of number of particles arriving at the substrate to those sputtered out of the target). The sputtering yield partially depends on the mass ratio between the incident ions and the target material. Increasing the ion mass can lead to a lower sputtering yield for light elements, while it can lead to a higher yield for heavy elements [27]. Meanwhile, sputtering with heavier ions may reduce the deposition probability since the thermalization distance also depends on the mass ratio between the gas atom and the target material [1], despite the lower sputtering pressure. The much lower growth rate of B_4_C would significantly prolong the deposition process of a real mirror with over 300 mm length, which may cause a larger thickness drift of the multilayer structure.

**Figure 3:**
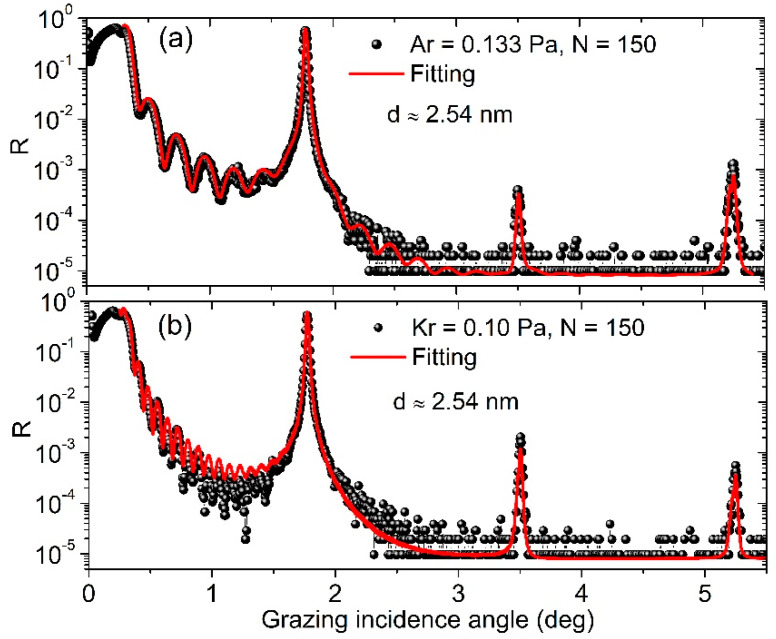
GIXR measurements and fitted results of the Ar-made (**a**) and Kr-made (**b**) multilayers with 150 bilayers.

The GIXR results of the 150-bilayer samples are shown in Figure 3. Both samples showed sharp Bragg peaks and the fitted period drift was approximately 40 pm. The drift was only slightly larger than the 50-bilayer multilayers discussed above, implying that the sputtering process became more stable after the initial stage. The peak reflectance of the first order was 55% for the Ar-made sample and 52% for the Kr-made sample, at *E* = 8.05 keV. The reflectance of Ar-made sample was similar to the maximum reported value in other works [18]. If the beam divergence of 0.007° was corrected, the reflectance would be further increased by 3–4%. The dense fringes after the total external reflection peak of the Kr-made sample were caused by the extra 20 nm-thick Si capping layer deposited on the top, which also induced a ~1% drop in reflectance. The fitted interface widths of the Ar- and Kr-made samples were 0.26 nm and 0.29 nm, respectively, which are almost the same as the multilayers with 50 bilayers. No accumulation of interface roughness was found.

**Figure 4:**
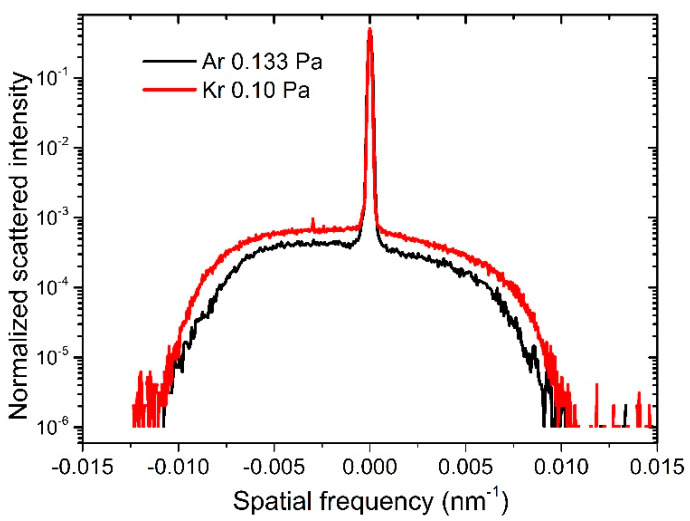
Rocking curve measurements of the 150-bilayer samples sputtered by two gases.

The rocking curve measurements of the two samples with 150 bilayers are shown in Figure 4. The spatial frequency was calculated as $\nu =\frac{1}{\lambda }\left|\cos\theta -\cos\theta_{0}\right|$, where *θ_0_* is the grazing incident angle, *θ* is the scattering angle with respect to the sample surface, and *λ* is the X-ray wavelength. It can be seen that the Kr-made sample had slightly higher scattering wings than the Ar-made sample, which means a larger interface roughness of the multilayer. This could have contributed to the larger interface width of the multilayer sputtered by Kr.

**Figure 5:**
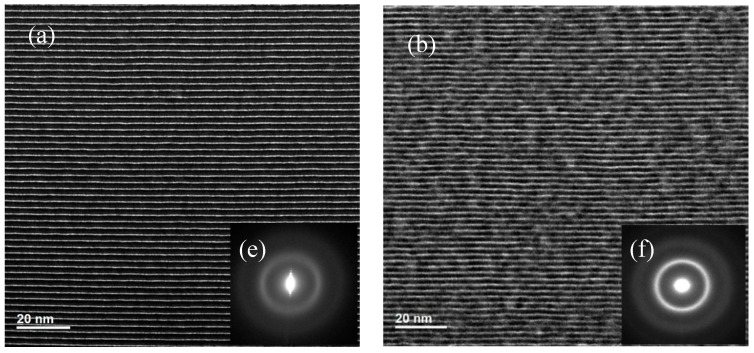

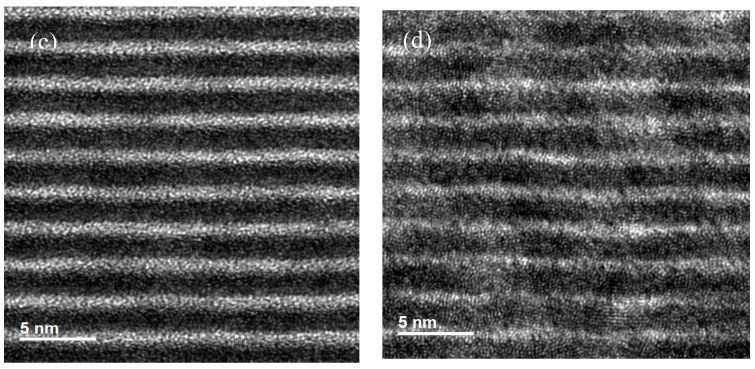
Transmission electron microscopy (TEM) and selected area electron diffraction (SAED) images of the 150-bilayer multilayers fabricated by Ar (**a**,**c**,**e**) and Kr (**b**,**d**,**f**).

The cross-sectional TEM images and selected area electron diffraction (SAED) patterns of the 150 bilayers samples are shown in Figure 5. The bright layers are B_4_C and the dark layers are Pd. The low-magnification images already exhibited flat layers with sharp interfaces for the sample made by Ar (Figure 5a), while the layer interfaces of the sample made by Kr are a little wavy (Figure 5b). In the high-resolution images, it can be seen that the Ar-made multilayer has an almost amorphous layer structure. On the contrary, the Kr-made multilayer has a polycrystalline structure with nanograins randomly distributed in the Pd layers. These nanograins have different sizes and growth orientations, which make the layer interfaces less sharp than the Ar-made multilayer [28]. This is consistent with the relatively large interface roughness of the Kr-made sample, as shown by the scattering measurements in Figure 4. The layer crystallization can be further seen in the SAED results. The Ar-made sample displayed a faint and broad ring, indicating an almost amorphous structure (Figure 5e). However, the small diffraction spots near the center transmission beam, which come from the diffraction from the periodic multilayer, are clear, indicating a good quality of the layer structure. The Kr-made sample showed significantly stronger diffraction rings (Figure 5f). Assuming that the compound formation of palladium boride is negligible in this multilayer system deposited at room temperature, the inner and outer rings can be identified as Pd(111) and Pd(200), respectively [13]. The phenomenon of stronger crystallization was also found in the Fe-based multilayers sputtered by Kr as compared to Ar [29,30].

**Figure 6:**
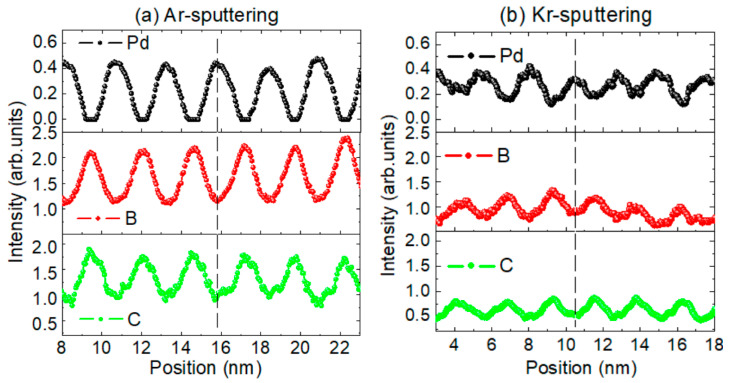
Electron energy loss spectroscopy (EELS) results of the depth profiles of the elemental concentration in Ar (**a**) and Kr (**b**) sputtered samples.

The depth profiles of the elemental composition of the two samples are shown in Figure 6. As the EELS signal cannot be properly normalized due to the limitations of the instrument, so only the signal intensity is shown. Both the profiles of Pd and B, C of the two samples exhibit periodic oscillation along the depth position, and the peak concentrations of Pd and B,C are in opposite positions. The amplitude of the oscillation of the Ar made sample is larger than that of the Kr-made sample, which implies a better elemental contrast of the Ar made multilayer. This is consistent with the larger interface width and lower optical contrast of the Kr-made sample found above. A residual amount of Ar atoms was found in the Ar made sample. Due to the influence of background noise in EELS measurement, it is not possible to determine whether Ar was in Pd or B_4_C layers. According to the energy-dispersive X-ray measurement, the atomic concentration of Ar reached approximately 7%. For the Kr-made sample, the Kr atoms were undetectable, which means the concentration was lower than 1%. The incorporation of the sputtered noble gas atoms can be mainly attributed to the recoiled gas particles. It has been reported that the energy and flux of the recoiled particles depend on the mass of the sputtering gas and target. Increasing the mass of the sputtering gas can reduce the energy and flux of the recoiled particles [12,22]. This explains the much lower concentration of Kr in the sputtered sample than Ar. According to the different characterization results discussed above, it can be seen that although the Kr sputtering process working at a lower pressure may bring a higher kinetic energy of the deposited atoms (particularly for Pd), which can contribute to smooth interfaces [12], the weaker bombardment from the fewer recoiled particles seemed to play a more important role in the growth of Pd/B_4_C multilayers, which eventually resulted in the increased interface roughness and crystallization as compared to the Ar made sample.

**Figure 7:**
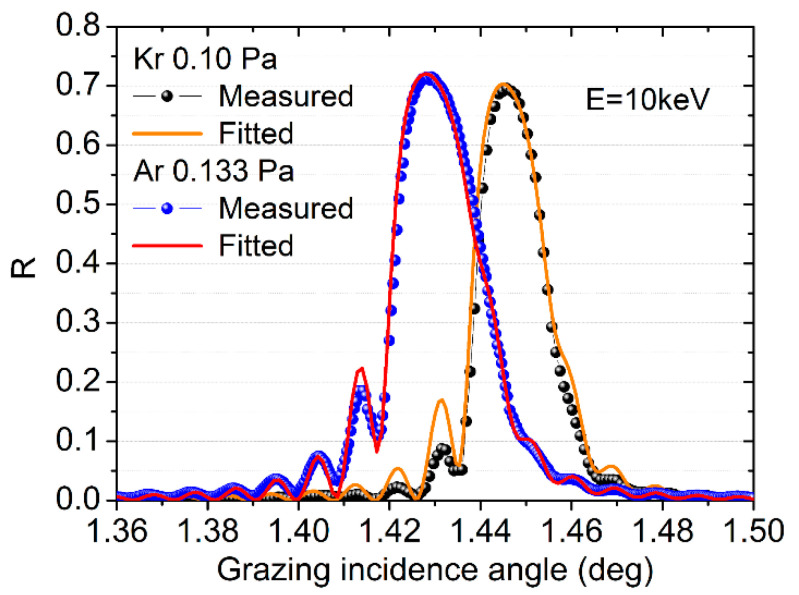
X-ray reflectance of the two multilayers with 150 bilayers measured in the Shanghai Synchrotron Radiation Facility (SSRF).

The X-ray reflectance results of the 150-bilayer samples measured at SSRF together with the fitted results are shown in Figure 7. A very high reflectance of 71% and 69% at 10 keV was demonstrated for the Ar and Kr made samples, respectively, and the Ar made sample showed a slightly higher value. The trend is consistent with the 8 keV reflectance measured in the lab. The fitted layer structures were almost the same as the one obtained from the GIXR measurements, which also verifies the results. The angular resolution, Δ*θ/θ* (where Δ*θ* is the full width at half maximum of the peak), was 1.5% and 1.2% for Ar and Kr made samples. The slightly narrower bandwidth of the Kr-made multilayer could be caused by the lower optical contrast (Figure 2) and the slightly different thickness ratio between the two samples. Considering the negligible content of the sputtering gas atoms inside the multilayer made by Kr and the relatively close reflectance, it could be advantageous to use Kr as the sputtering gas if the absorption edges or fluorescence signals of the gas atoms need to be avoided in applications [31].

## 4. Conclusions

Pd/B_4_C multilayers fabricated by Kr and Ar gases were studied comparatively. Although the sputtering process using the heavier gas of Kr can work at a lower pressure, the interface widths, particularly the interface roughness, were slightly increased and the optical contrast of the layers was reduced. The multilayers with 50 and 150 bilayers showed almost the same layer structures and no accumulation of roughness was found for either process. Stronger polycrystallization of the Pd layers was found in the Kr-sputtered sample, which could have contributed to the larger roughness. A significantly higher content of sputtering gas atoms was found in the Ar-made sample, while the content in the Kr-made sample was close to zero. This can be explained by the much lower amount and lower energy of the recoiled heavy particles of Kr. The different effects induced by using a heavier gas (i.e., less bombardment of the layers from the recoiled particles and the possibly higher kinetic energy of the deposited atoms from the lower working pressure) balanced each other, and resulted in the different microstructure of the multilayers. The Kr sputtering process also greatly reduced the growth rate of B_4_C layers, while its related effect on the B_4_C layer structure needs more analysis and characterization. Despite the slightly worse layer structure of the Kr-made sample, the impact on the hard X-ray reflectance was minor. High reflectances of 71% and 69% were demonstrated at 10 keV for Ar- and Kr-made multilayers, respectively. Given to the rapid degradation of ultrathin Pd/B_4_C multilayers stored in air [32], the lifetime and stability of multilayers fabricated by Kr need to be further studied. The intrinsic stress of the multilayers fabricated by different gases is also worth comparison. The properties of the Kr-sputtered Pd/B_4_C multilayer and the related characteristics of the process found in this work can provide useful guidance for the development of other X-ray multilayers and thin films using gases heavier than Ar.

## Figures and Tables

**Table 1:** Fitted layer structure parameters of the Pd/B_4_C multilayers with 50 bilayers.

	Materials	Average Layer Thickness (nm)	d-Spacing Drift (pm)	Average Interface Width (nm)
Kr 0.10 Pa	Pd	1.320	25	0.30
	B_4_C	1.247		
Ar 0.133 Pa	Pd	1.337	30	0.26
	B_4_C	1.252		
Ar 0.10 Pa	Pd	1.246	60	0.27
	B_4_C	1.460		
